# Validity of Field Methods to Estimate Fat-Free Mass Changes Throughout the Season in Elite Youth Soccer Players

**DOI:** 10.3389/fphys.2020.00016

**Published:** 2020-02-12

**Authors:** F. Javier Núñez, Diego Munguía-Izquierdo, Luis Suárez-Arrones

**Affiliations:** Physical Performance and Sports Research, Pablo de Olavide University, Seville, Spain

**Keywords:** body composition, DXA, team game, indirect methods, sensitivity to change

## Abstract

The aim of this study was to determine the most effective anthropometric equations or bioelectrical impedance analysis (BIA) devices for quantifying the sensitivity to change in fat-free mass (FFM) in elite young soccer players, in comparison with measurements using dual-energy X-ray absorptiometry (DXA), between the pre- and mid-season. A total of 40 elite youth soccer players participated in this study. DXA values provided a criterion measure of FFM. Correlation coefficients, biases, limits of agreement, and differences were used as measures of sensitivity to change. All body density, skinfold, and anthropometric equations and BIA devices used to obtain FFM data showed positive and very large correlations (*r* from 0.70 to 0.89) with DXA. A significant increase in FFM was shown between time points using DXA, BIA, and all anthropometric equations (*p* < 0.01). The magnitudes of differences were small for DXA, BIA inbody and all anthropometric equations except those of Faulkner (1966), Durnin and Rahaman (1967), Brook (1971), and Sarría et al. (1998). Six anthropometric equations [Faulkner (1966), Durnin and Womersley (1974), Carter (1982), Slaughter et al. (1988), Reilly et al. (2009), and Munguia-Izquierdo et al. (2018)] and BIA Tanita showed no statistical differences compared to DXA, with a low bias. We concluded that the equations developed by Durnin and Womersley (1974), Carter (1982), Slaughter et al. (1988), Reilly et al. (2009), and Munguia-Izquierdo et al. (2018) showed the best sensitivity in assessing FFM changes between pre- and mid-season in elite youth soccer players.

## Introduction

Body composition is a key fitness element relevant to soccer players’ performance and to the risk of injury (Suarez-Arrones et al., 2018) especially in young soccer players (Munguia-Izquierdo et al., 2018). Of the different body composition components of young soccer players, fat-free mass (FFM) strongly contributes to strength and power performance (Deprez et al., 2015). In fact, FFM is considered the best longitudinal predictor for leg power during late adolescence in young soccer players (Milsom et al., 2015), and the routine evaluation of FFM throughout the season provides useful information for coaches and nutritionists to monitor the efficacy of training and nutrition (Suarez-Arrones et al., 2019).

A range of methods is used for the evaluation of FFM. Dual-energy X-ray absorptiometry (DXA) is one of the most reliable techniques for assessing FFM in young soccer players (Nana et al., 2016; Munguia-Izquierdo et al., 2018). However, DXA is costly and cannot be used frequently (Sutton et al., 2009). Therefore, it is usual to use lower cost methods, such as bioelectrical impedance analysis (BIA) and anthropometric measurements (circumference, skinfold, and breadth), to estimate FFM (Nana et al., 2016). A recent study compared a variety of field methods with DXA for the assessment of FFM in elite youth male soccer players (Munguia-Izquierdo et al., 2018) and showed that only the body density equations proposed by Durnin and Womersley (1974) and Sarría et al. (1998), the skinfold equation proposed by Slaughter et al. (1988), and the new anthropometric soccer-specific equation proposed by Munguia-Izquierdo et al. (2018) were suitable for the cross-sectional assessment of FFM in young soccer players (Munguia-Izquierdo et al., 2018).

Mechanical and physiological stresses during training sessions and competitive matches can modulate players’ body composition throughout the season (Milanese et al., 2015; Milsom et al., 2015; Devlin et al., 2017). A recent study showed that a strength-training program that supplements soccer-related training sessions in young professional soccer players may be an effective option to increase FFM, assessed in this case by DXA (Suarez-Arrones et al., 2019). The typical evaluation of FFM in young soccer involves the use of anthropometric measures or BIA using regression equations based on a cross-sectional relationship between anthropometric or BIA variables and DXA (Munguia-Izquierdo et al., 2018). A study with rugby league players showed that the lean-mass index [developed by Slater et al. (2006), using the sum of seven skinfold measurements, body mass, and two different exponents] was very likely to be more useful than BIA, and possibly a better estimate of FFM changes than the anthropometric formula by Slater et al. (2006) for FFM (Delaney et al., 2016). However, the ability of these practical estimates to detect changes in FFM during a season needs to be determined in team sports athletes with different somatotypes, such as soccer players. Although different anthropometric equations and BIA are widely used as practical and non-invasive alternative to DXA to predict FFM changes in young soccer players, no data are currently available about the sensitivity to change of these methods in elite young soccer players during a season. Consequently, the aim of this study was to determine the most effective field method for quantifying FFM sensitivity to change in elite young soccer players against DXA, between pre- and mid-season.

## Materials and Methods

### Subjects

Forty male elite-standard youth soccer players were recruited for this study from the development program of a professional Spanish soccer club competing in La Liga. The physical characteristics of the participants are shown in Table 1. Data were collected during a season where all players performed four to five regular training sessions per week (about 90–120 min per session) and played one official soccer game per week. Before beginning the study, written consent was obtained from the participants and their parents or legal representatives after they had been fully informed about the experimental procedures, purpose, and potential risks of the study.

**TABLE 1:** Physical characteristics of the participants (*n* = 40).

	Pre-season	Mid-season
Age (years)	16.67 ± 0.5	17.07 ± 0.5
Weight (kg)	66.65 ± 5.2	67.68 ± 5.4
Height (cm)	174 ± 5	175 ± 5
BMI (kg/m^2^)	21.91 ± 1.2	22.06 ± 1.2
% fat mass by DXA	11.86 ± 2.5	11.31 ± 2.5
**Skinfolds (mm)**		
Triceps	7.9 ± 1	8.3 ± 2
Subscapular	7.9 ± 1	7.9 ± 1
Biceps	3.5 ± 0.6	3.2 ± 0.4
Iliac rest	11 ± 3.5	11.1 ± 3.6
Supraspinale	6.5 ± 1.7	6.8 ± 1.9
Abdominal	10 ± 2.8	9.7 ± 3
Anterior thigh	11.5 ± 2.8	10.6 ± 2.6
Medial calf	7.9 ± 2.6	7 ± 2.1
**Circumferences (cm)**		
Arm relaxed	26.9 ± 1.6	26.9 ± 1.6
Arm flexed	30.1 ± 1.7	30.5 ± 1.8
Waist	74.5 ± 2.7	74.8 ± 2.6
Hip	91.6 ± 3.4	92.5 ± 3.6
Thigh	35.4 ± 1.4	35.8 ± 1.3
**Breadths (cm)**		
Humerus	6.9 ± 0.4	6.9 ± 0.3
Femur	9.7 ± 0.4	9.7 ± 0.3

*Data are mean ± SD. BMI, body mass index; %BF, percentage of body fat; FFM, fat-free mass; DXA, X-ray absorptiometry.*

### Procedure

This was a cross-sectional validation study in which the height, body mass, FFM (BIA), skinfolds, circumferences, breadths, and FFM (using DXA) of the participants were measured using standardized procedures in the aforementioned order. The FFM estimations from BIA measurements and anthropometric equations during the pre-season (August–September) and during the mid-season (January–February) were compared with DXA results to determine the sensitivity to change of these practical methods for use in elite youth soccer players. Participants were instructed to follow an identical assessment session in the same well-ventilated room with controlled temperature and humidity following standard food and fluid protocol so they presented in a rested, overnight fasted, and hydrated state before testing. They were also asked to arrive with an empty bladder and bowel (Yamada et al., 2017). Subjects wore only shorts during the evaluation and removed any metal and jewelry before assessment. They were asked to avoid strenuous exercise, alcohol, stimulants, or depressants for 24 h before testing (Munguia-Izquierdo et al., 2018). Each participant undertook an identical assessment session for 30–45 min on different days.

#### Bioelectrical Impedance Analysis

Before BIA measurement, the participants’ palms and the soles of their feet were wiped with an electrolyte tissue. The BIA measurements were taken using InBody 770 (Biospace, Seoul, South Korea) and Tanita BC-418 (Tanita Corp., Tokyo, Japan) devices. Body mass was measured with the players standing and with their feet in contact with the foot electrodes of the InBody 770 and Tanita BC-418 scales. Participants grasped the hand grips, placing their fingers in the standard locations. Analysis of FFM started when the participant was immobile in the position described. Impedance measurements were performed three times with each of the InBody 770 and Tanita BC-418 devices for each participant, and the mean of the two first FFM measures was used for analysis. If the two first measures differed by more than 0.05 kg, the third measure was included, and the median of the triplicate measurements was used for subsequent analysis.

#### Anthropometry

Stature was measured using a stadiometer (Seca 711, Hamburg, Germany) with an accuracy of 0.5 cm. Body mass was measured with an electronic scale (OHAUS Corp., Florham Park, NJ, United States) with an accuracy of 0.1 kg. The formula for the body mass index (BMI) was: body mass (kg)/height (m^2^). Six circumferences (calf, thigh, waist, hip, arm relaxed, and arm flexed), eight skinfold thicknesses (medial calf, anterior thigh, iliac crest, abdominal, subscapular, supraspinal, biceps, and triceps), and two bone biepicondylar breadths (femur and humerus) were measured with a tape, skinfold caliper, and caliper (Holtain, Crymych, United Kingdom), respectively. All anthropometric measurements were taken according to standard methods (Marfell-Jones et al., 2012). The mean of two measures of each anthropometric variable was used for analysis. A third measure was taken if the technical error of measurement was exceeded (> 5% for skinfold measurements and >1% for circumferences and bone breadths), and the median of the triplicate measurements was then used for subsequent analysis. Twelve equations were used to estimate the percentage of body fat (Faulkner, 1966; Durnin and Rahaman, 1967; Brook, 1971; Durnin and Womersley, 1974; Lohman, 1981; Carter, 1982; Withers et al., 1987; Deurenberg et al., 1991; Sarría et al., 1998; Reilly et al., 2009; Munguia-Izquierdo et al., 2018). The Siri equation (Suarez-Arrones et al., 2018) was used to estimate the percentage of body fat, when the body density was calculated from an equation. Fat mass was subtracted from body mass to obtain the FFM (0.1 kg). In addition, a new soccer-specific equation (Munguia-Izquierdo et al., 2018) was used to estimate FFM.

#### Dual-Energy X-ray Absorptiometry

A DXA scanner (Hologic Corp., Hologic Series Discovery QDR, Bedford, MA, United States) with software (Physician’s Viewer, APEX System Software Version 3.1.2, Bedford, MA, United States) was used to calculate FFM, following the DXA best practice guidelines described previously (Nana et al., 2016). Before any measurements, the DXA was calibrated each day with phantoms, as per the manufacturer’s guidelines. The participants were in a supine position with their hands level with their hips and their feet slightly apart. All DXA scans, which were completed with the same device and software, were performed by at least one of the two certified and trained DXA technicians, who positioned the participants, performed the scan, and executed the analysis in routine clinical manner following research facility standard operating procedures. All scans were analyzed using the software autoanalysis feature followed by manual correction of analysis markers when necessary to avoid measurement errors. Total body and regional analyses were performed in a routine clinical manner. Six standard regions of interest were used: total body, trunk, right arm, left arm, right leg, and left leg. FFM was obtained from the sum of the lean soft tissue mass and bone mineral content and was calculated from whole-body scans excluding the head. The FFM coefficient of variation for repeated measures was <3.3% (Calbet et al., 1998).

### Statistical Analyses

Descriptive statistics were calculated for each variable. The normality of the distribution of the data was verified using the Shapiro–Wilk test. To compare FFM sensibility to change between DXA, BIA data, and anthropometric equations, paired *t* tests, Pearson’s correlation (±90% CI), bias, limits of agreement, and standardized differences (±90% CI) were used. Correlation coefficients were qualitatively ranked by magnitude as follows: trivial, *r* < 0.1; small, 0.1 < *r* < 0.3; moderate, 0.3 < *r* < 0.5; large, 0.5 < *r* < 0.7; very large, 0.7 < *r* < 0.9; almost perfect, 0.9 < *r* < 1.0; and perfect *r* = 1.0 (Hopkins et al., 2009). The effect size of the standardized differences in FFM was determined using Cohen’s *d* statistic, and Hopkins’ scale was used to determine the magnitude of the effect size, where 0–0.2 = trivial, 0.2–0.6 = small, 0.6–1.2 = moderate, 1.2–2.0 = large, and >2.0 = very large (Hopkins et al., 2009).

## Results

The technical errors of measurement of all the anthropometric measurements performed were lower than the standards indicated by the International Society for the Advancement of Kinanthropometry, as shown in a previous study conducted with the same sample (Munguia-Izquierdo et al., 2018). Table 2 shows the magnitude of the changes between pre- and mid-season with DXA and other practical estimates of FFM. A significant increase in FFM was shown between time points using DXA, BIA, and all anthropometric equations (*p* < 0.01). The magnitudes of the differences were small for DXA, BIA inbody, and all anthropometric equations except those of Faulkner (1966), Durnin and Rahaman (1967), Brook (1971), and Sarría et al. (1998).

**TABLE 2:** Change in estimation of FFM with DXA and other practical estimates of FFM between the pre- and mid-season.

Variable	Pre-Season	Mid-Season	% Diff (CL)	ES (CL)
DXA FFM	55.73 ± 4.04	56.79 ± 4.15	1.91 (1.19;2.63)*	0.26 (0.16;0.35)
Slaughter et al.	58.08 ± 4.03	59.31 ± 4.21	2.11 (1.49;2.72)*	0.30 (0.21;0.38)
Faulkner	59.45 ± 4,45	60.33 ± 4,61	1.48 (0.83;2.12)*	0.19 (0.11;0.28)
Carter	61.26 ± 4.55	62.31 ± 4.77	1.70 (1.05;2.35)*	0.22 (0.14;0.31)
Durnin-Womersley	58.10 ± 4.22	59.40 ± 4.36	2.23 (1.64;2.84)*	0.30 (0.22;0.38)
Durnin-Rahaman	56.02 ± 3.91	60.19 ± 4.35	7.43 (6.77;8.09)*	1.01 (0.92;1.10)
Brook	55.24 ± 3.79	55.94 ± 3.95	1.25 (0.68;1.82)*	0.18 (0.10;0.26)
Withers et al.	60.08 ± 4.30	61.89 ± 4.57	3.01 (2.34;3.68)*	0.41 (0.32;0.50)
Lohman	61.88 ± 4.68	63.56 ± 4.89	2.70 (1.98;3.43)*	0.35 (0.26;0.44)
Sarria et al.	59.20 ± 4.14	60.00 ± 4.30	1.33 (0.75;1.91)*	0.19 (0.11;0.27)
Deurenberg et al.	58.74 ± 4.27	60.25 ± 4.49	2.57 (1.89;3.25)*	0.34 (0.25;0.43)
Reilly et al.	59.50 ± 4.38	60.57 ± 4.59	1.78 (1.13;2.43)*	0.24 (0.15;0.32)
Lean mass index	38.81 ± 2.71	39.59 ± 2.86	1.97 (1.40;2.54)*	0.28 (0.20;0.35)
Munguia-Izquierdo et al.	58.45 ± 4.21	59.81 ± 4.37	2.32 (1.48;3.17)*	0.31 (0.20;0.43)
BIA inbody	57.09 ± 4.61	58.97 ± 4.77	3.50 (2.63;4.37)*	0.42 (0.31;0.52)
BIA Tanita	56.31 ± 4.24	57.09 ± 4.38	1.38 (0.74;2.03)*	0.18 (0.10;0.26)

Data are mean ± SD. *Significant differences between the pre- and mid-season using paired *t* test (*p* < 0.01). CL, confidence limits; ES, effect size.

Correlations, biases, limits of agreement, and standardized differences between changes in FFM with DXA and other practical estimates in elite youth soccer players are shown in Table 3. All body density, skinfold, and anthropometric equations and BIA FFM data showed positive and very large correlations (*r* from 0.70 to 0.89) with DXA. Only BIA Tanita and one set of equations showed no standardized or substantial differences against DXA and had the lowest bias (Faulkner, 1966; Durnin and Womersley, 1974; Carter, 1982; Slaughter et al., 1988; Reilly et al., 2009; Munguia-Izquierdo et al., 2018).

**TABLE 3:** Correlations, biases, limits of agreement, and standardized differences between changes in FFM with DXA and other practical estimates in elite youth soccer players (*n* = 40).

Estimates of fat-free mass	Correlation (90% CI)	Bias (±LoA)	Standardized differences (90% CL)
Slaughter et al.	0.87 (0.79;0.92)	0.16(±1.40)	0.02 (0.40)
Faulkner	0.86 (0.77;0.91)	−0.18(±1.50)	−0.09(0.39)
Carter	0.86 (0.77;0.92)	−0.02(±1.52)	−0.09(0.39)
Durnin-Womersley	0.82 (0.71;0.89)	0.23(±1.65)	0.19 (0.33)
Durnin-Rahaman	0.79 (0.66;0.87)	3.10(±1.81)	3.13 (0.74)**
Brook	0.79 (0.67;0.87)	−0.37(±1.74)	−0.51 (0.36)*
Withers et al.	0.85 (0.76;0.91)	0.75(±1.57)	0.14 (0.36)**
Lohman	0.82 (0.72;0.89)	0.61(±1.84)	0.19 (0.37)**
Sarria et al.	0.83 (0.72;0.90)	−0.27(±1.60)	−0.39 (0.35)*
Deurenberg et al.	0.82 (0.71;0.89)	0.45(±1.73)	0.18 (0.41)*
Reilly et al.	0.88 (0.80;0.93)	0.00(±1.41)	0.01 (0.39)
Lean mass index	0.89 (0.81;0.93)	−0.29(±1.62)	−0.41 (0.38)*
Munguia-Izquierdo et al.	0.75 (0.61;0.85)	0.29(±2.31)	0.09 (0.36)
BIA inbody	0.70 (0.53;0.82)	0.87(±2.54)	0.58 (0.41)**
BIA Tanita	0.78 (0.64;0.86)	−0.28(±1.85)	−0.14(0.39)

Significant differences between criterion (DXA) fat-free mass and others practical estimates of fat-free mass using paired *t* test *(*p* < 0.05), **(*p* < 0.01). CI, confidence interval; LoA, level of agreement; CL, confidence level; BIA, bioelectrical impedance analysis.

## Discussion

The aim of this study was to determine the most effective field method to estimate FFM changes in elite young soccer players. The main finding of this study was that the equations developed by Durnin and Womersley (1974), Carter (1982), Slaughter et al. (1988), Reilly et al. (2009), and Munguia-Izquierdo et al. (2018) were accurate, highly correlated with DXA, and showed lower biases in estimating FFM changes in elite youth soccer players.

Anthropometric equations produced by Durnin and Womersley (1974), Carter (1982), Slaughter et al. (1988), Reilly et al. (2009), and a new soccer-specific equation developed by Munguia-Izquierdo et al. (2018) were similar predictors of DXA-derived FFM changes between pre- and mid-season. To the author’s knowledge, no previous investigation has analyzed these field methods to estimate FFM changes throughout the season in elite youth soccer players. A previous study with rugby league players showed that the lean-mass index was very likely to be more useful than BIA for a better estimate of FFM changes. A recent study with elite youth soccer players also showed that five anthropometric equations were more useful than BIA for estimating of FFM changes. Lean-mass index and BIA (inbody) showed statistical differences in biases in comparison with DXA (Munguia-Izquierdo et al., 2018). The equations of Durnin and Womersley (1974) and Slaughter et al. (1988) were developed from data collected from more heterogeneous samples. However, the equation developed by Munguia-Izquierdo et al. (2018) was specific for this population and had similar validity results to the other two equations. The equations proposed by Reilly et al. (2009) were developed with professional soccer players and showed the lowest bias of all, but did not show the highest accuracy in estimating FFM in youth soccer players (2018). The five equations showed very large correlations with DXA, and the biases indicate that these should be used for estimating FFM changes between pre- and mid-season in youth soccer players.

The FFM increase found between pre- and mid-season using DXA in the present investigation (+1.9%) was slightly higher than that found by Milanese et al. (2015) in professional soccer players (+1.5%). These differences can be explained by multiple factors such as the age of the players, the training that these players carried out over that period, and the number of matches played. In addition, our sample used outfield players, and Milanese et al. (2015) used outfield players and four goalkeepers. There is evidence of FFM positional differences between goalkeepers and outfield players (Milsom et al., 2015), but no differences between specific positions were apparent when considering FFM in outfield players (Milanese et al., 2015; Milsom et al., 2015). Our results were similar to those obtained by Devlin et al. (2017) during the pre-season in professional soccer players (+1.9%). These authors showed that fat-free soft tissue mass increased from the start of the pre-season to the start of the competitive season, and these gains were maintained until the end of the season (2017). Nevertheless, our results were lower than those obtained by Suarez-Arrones et al. (2019) in young male soccer players after combined soccer and strength training (+5.1%). These differences can be explained because Suarez-Arrones et al. (Suarez-Arrones et al., 2019) used a specific strength training program that previously produced FFM changes in professional soccer players, and the period examined was a whole season.

The present study had several limitations. We instructed the participants to arrive in the same standardized condition during the pre- and mid-season measurements to minimize the influence of the hydration on the determination of FFM; however, the lack of a hydration status measurement before the FFM assessment was a limitation of the current study. Further studies assessing urine-specific gravity of all players are needed to ensure adequate hydration before each assessment. Several studies have showed that FFM shows greater increases from the start of the pre- to mid-season and lower increases from mid- to end of season (Devlin et al., 2017; Owen et al., 2018). To quantify the FFM sensitivity to change, we used the first period rather than the second.

## Conclusion

BIA and all anthropometric equations used in this study to estimate FFM change showed high correlations with DXA data, but the equations developed by Durnin and Womersley (1974), Carter (1982), Slaughter et al. (1988), Reilly et al. (2009), and Munguia-Izquierdo et al. (2018) showed the best sensitivity in assessing FFM change between pre- and mid-season in elite youth soccer players.

## Data Availability Statement

The datasets generated for this study are available on request to the corresponding author.

## Ethics Statement

The studies involving human participants were reviewed and approved by the Pablo Olavide University ethics committee. Written informed consent to participate in this study was provided by the participants’ legal guardian/next of kin.

## Author Contributions

All authors listed have made a substantial, direct and intellectual contribution to the work, and approved it for publication.

## Conflict of Interest

The authors declare that the research was conducted in the absence of any commercial or financial relationships that could be construed as a potential conflict of interest.
